# Design of a Seed-Specific Chimeric Promoter with a Modified Expression Profile to Improve Seed Oil Content

**DOI:** 10.3390/ijms19061667

**Published:** 2018-06-05

**Authors:** Toshihiro Aoyagi, Masaya Kobayashi, Akiko Kozaki

**Affiliations:** Department of Biology, Shizuoka University, 836 Ohya Suruga-ku, Shizuoka 422-8529, Japan; sakozak42@gmail.com (T.A.); kozakiakiko@icloud.com (M.K.)

**Keywords:** triacyl glycerol, seed oil, chimeric promoter, diacylglycerol acyltransferase 1, *Arabidopsis*, biotin carboxyl carrier protein 2, fatty acid elongase 1, WRINKLED 1

## Abstract

Increasing the yield of plant oil is an important objective to meet the demand for sustainable resources and energy. Some attempts to enhance the expression of genes involved in oil synthesis in seeds have succeeded in increasing oil content. In many cases, the promoters of seed-storage protein genes have been used as seed-specific promoters. However, conventional promoters are developmentally regulated and their expression periods are limited. We constructed a chimeric promoter that starts to express in the early stage of seed development, and high-level expression is retained until the later stage by connecting the promoters of the *biotin carboxyl carrier protein 2* (*BCCP2*) gene encoding the BCCP2 subunit of acetyl-CoA carboxylase and the *fatty acid elongase 1* (*FAE1*) gene from *Arabidopsis*. The constructed promoter was ligated upstream of the *TAG1* gene encoding diacylglycerol acyltransferase 1 and introduced into *Arabidopsis*. Seeds from transgenic plants carrying *AtTAG1* under the control of the chimeric promoter showed increased oil content (up by 18–73%) compared with wild-type seeds. The novel expression profile of the chimeric promoter showed that this could be a promising strategy to manipulate the content of seed-storage oils and other compounds.

## 1. Introduction

Many plants accumulate storage reserves such as oil, carbohydrate and protein in seeds, and these reserves are important not only for growth of juvenile seedlings but also for human nutrition and as a source of chemicals for industry. Among these storage reserves, the demand for vegetable oils as fuels and chemical resources has increased because there is a need to increase the use of sustainable energy and chemical resources. However, the yield of plant oil is not enough to meet this demand. Therefore, there is an urgent need to improve the yield of oil crops.

The relative proportions of oil, carbohydrate and protein vary depending on the plant species. The accumulation of each class of storage component requires the coordination of many genes that encode the enzymes in the respective pathways [1,2]. Therefore, it is important to reveal the regulation of these genes involved in the metabolism of these storage components to understand how the carbon resources in seeds are allocated to each storage component.

During early embryogenesis, carbon and other nutrients are used mainly for rapid cell division and embryo growth. After cell division ceases, during the maturation phase, the resources are allocated to synthesize storage compounds. In *Arabidopsis*, starch accumulates transiently in the early stage of the maturation phase, followed by a major increase in oil and protein contents. The transiently accumulated starch disappears later and presumably is used as a carbon source for fatty acid and protein synthesis. During the subsequent late maturation and desiccation phases, the overall biosynthetic activity decreases as the seed prepares for dormancy [3].

The temporal patterns of gene expression reflect the accumulation of storage compounds. In the early stage of *Arabidopsis* seed development, transcripts for starch synthesis are increased transiently and then reduced to low levels by 8–10 days after flowering (DAF). On the other hand, transcripts for fatty acid synthesis increase gradually from the early stage to maximum level at 10–12 DAF and then start to decrease before those for storage protein synthesis. Transcripts for protein synthesis start to increase at ~8 DAF, and high transcript levels are retained at 13 DAF [4]. Because carbon resources are limited in developing seeds, there is probably competition for these between fatty acid synthesis and protein synthesis.

Seed storage oils are synthesized in the form of triacylglycerol (TAG). In plants, TAG synthesis occurs in different subcellular compartments. Fatty acids are synthesized in plastids and then exported to the cytosol and activated to form acyl-CoA, which serves as the substrate for the esterification of glycerol-3-phosphate at the endoplasmic reticulum to form glycerolipids. The expression of TAG synthesis genes can be characterized by two main expression patterns in *Arabidopsis* [4]. The expression of many genes for fatty acid synthesis followed a bell-shaped pattern that increased during early developmental stages, peaked between eight and 12 DAF, and then decreased as mentioned previously. The first committed step of fatty acid synthesis is catalyzed by acetyl-CoA carboxylase (ACCase). Most plants, except for Gramineae, have a heteromeric form of ACCase in plastids, and the expression of genes for subunits of the heteromeric ACCase, such as biotin carboxyl carrier protein 1 (BCCP1), BCCP2 and biotin carboxylase, shows a bell-shaped pattern during seed development. On the other hand, the expression patterns of genes for enzymes that function outside of the plastids and are involved in the modification of fatty acids, such as *FAD3* encoding linoleate desaturase and *FAE1* encoding fatty acid elongase 1, show a different profile. Their expression starts to increase at ~8 DAF with maximum expression later in development, and a high level of expression is retained at 13 DAF. Recent research revealed that many of the genes for fatty acid synthesis are regulated by the transcription factor WRINKLED 1 (WRI1) [5,6].

To date, there have been several trials to manipulate plant oil content and composition [7,8]. In order to increase oil content in seeds, genes for enzymes involved in oil synthesis, such as cytosolic acetyl-CoA carboxylase (homomeric ACCase), lysophosphatidic acid acyltransferase and diacylglycerol acyltransferase 1 (DGAT1), have been overexpressed in seeds and have succeeded in enhancing seed oil content [9,10,11,12]. Several genes have also been manipulated simultaneously to further increase seed oil content [13,14,15].

In this article, we tried to construct a suitable promoter to express genes involved in oil synthesis to increase seed oil content. We suspect that the bell-shaped expression pattern of genes for fatty acid synthesis is one of the limiting factors for oil content in seeds, because the expression of genes for protein synthesis continued to increase after that of fatty acid synthesis genes was reduced. Therefore, we constructed a promoter that started to express at an early stage of seed development, and high-level expression was kept for later in development by connecting the promoters of the *AtBCCP2* gene encoding the BCCP2 subunit of acetyl-CoA carboxylase (ACCase) and the *AtFAE1* gene. The constructed promoter was ligated upstream of the *AtTAG1* gene encoding DGAT1 and introduced into *Arabidopsis*. Seeds from the transgenic plants carrying the *AtTAG1* gene under the control of the chimeric promoter showed higher oil content (up by 18–73%) compared with that of the wild-type (WT).

## 2. Results

### 2.1. Construction of the Chimeric Promoter

Previously, our research showed that the 5′-untranslated region (UTR) and 273-bp-upstream sequence from the transcriptional start site (TSS) was enough for *AtBCCP2* gene expression in seeds [16]. The 5′-UTR contains two WRI1 binding sites (AW box) and was strongly induced by WRI1 [17]. The *AtBCCP2* gene shows seed-specific expression, and the expression starts from the early stage of seed development, peaks at 10–12 DAF, and then decreases [18]. The *FAE1* promoter has been used as a seed-specific promoter. The 1-kb-upstream region from the translation initiation site (TIS) is sufficient for *AtFAE1* expression [19]. Its expression starts to increase slightly later than that of the *AtBCCP2* gene, at ~8 DAF, increases during seed maturation, and decreases after maturation [4,16].

**Figure 1:**
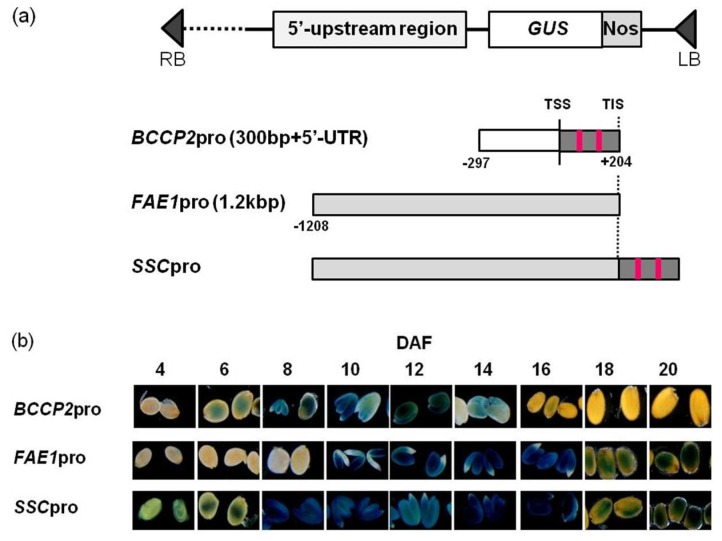
β-glucuronidase (*GUS*) expression analysis of transgenic plants containing the chimeric promoter. (**a**) Schematic diagram of the promoter: *GUS* constructs analyzed. The pink bars indicate the location of the AW box (WRI1 binding sequence). TIS: translational initiation sites. TSS: transcriptional start sites. LB: left border. RB: right border; (**b**) Histochemical staining of seeds at 4, 6, 8, 10, 12, 14, 16, 18 and 20 days after flowering (DAF) from plants carrying *BCCP2pro:GUS*, *FAE1pro:GUS* and *SSCpro:GUS*. For staining, seeds from #4 (*BCCP2pro:GUS*), #1 (*FAE1pro:GUS*) and #7 (*SSCpro:GUS)* in Figure 2 were used.

To construct a promoter that was expressed from the early stage of seed development to the later stage of seed maturation, we made a chimeric promoter that contained the regulatory regions of both *AtBCCP2* and *AtFAE1*. Because previous data indicated that the WRI1 binding sequence should be close to the transcription start site [16], the 1.2-kb-upstream region from the TIS of *AtFAE1* was ligated to the 5′ site of the *AtBCCP2* gene. Because the fragment of *FAE1* promoter contains the core promoter and the 5′-UTR of the *AtBCCP2* gene contains two AW boxes, the upstream region from the TSS of *AtBCCP2* gene was not used for construction of the chimeric promoter. We named the promoter as *seed-specific chimeric* (*SSC*) promoter (Figure 1a).

### 2.2. The Chimeric Promoter Shows Prolonged and Enhanced Activity during Seed Development

In order to examine the promoter activity, we constructed a reporter vector containing the β-glucuronidase (*GUS*) gene ligated downstream of the *SSC* promoter (Figure 1a). The *SSC* promoter (*SSCpro*):*GUS* gene was introduced into *Arabidopsis* and we obtained several transgenic plants. We stained the developing seeds at 4, 6, 8, 10, 12, 14, 16, 18 and 20 DAF. GUS staining was observed from 4 to 20 DAF, and the strongest staining was observed from 8 to 12 DAF (Figure 1b). To compare the promoter activity, we also made transgenic plants containing the 500-bp-upstream sequence from the TIS of the *AtBCCP2* gene fused with the *GUS* gene (*BCCP2pro:GUS*) and a 1.2-kb-upstream sequence from the TIS of the *AtFAE1* gene fused with *GUS* gene (*FAE1pro:GUS*), and stained the developing seeds from these transgenic plants (Figure 1). The staining patterns indicated that the period of the expression of the *SSC* promoter was the sum of those of the *BCCP2* and *FAE1* promoters (Figure 1b).

**Figure 2:**
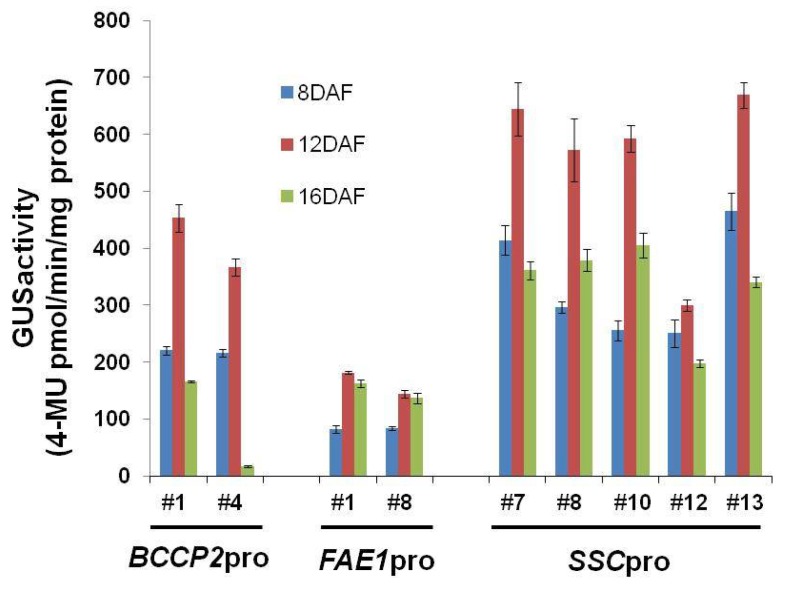
β-glucuronidase (*GUS*) activity in seeds at 8, 12 and 16 days after flowering (DAF). Quantitative analysis of β-glucuronidase (*GUS*) activity in seeds at 8, 12 and 16 days after flowering (DAF) from transgenic plants containing the biotin carboxyl carrier protein 2 (*BCCP2*), *fatty acid elongase 1* (*FAE1*) and the *seed-specific chimeric* (*SSC*) promoters. Values represent means of three independent experiments. Statistical analysis carried out by Student’s *t*-test. Error bars represent standard deviation.

To estimate the strength of the promoter, we measured the GUS activity of the transgenic plants. The GUS activities in seeds at 8, 12 and 16 DAF from *SSCpro:GUS*, *BCCP2pro:GUS* and *FAE1pro:GUS* plants were measured. The GUS activity of seeds from the *SSCpro:GUS* plants was higher than each activity of *BCCP2pro:GUS* and *FAE1pro:GUS* plants, and appeared to be the sum of each activity (Figure 2). The results showed that the *SSC* promoter was strongly expressed over the long term for seed development.

### 2.3. Oil Accumulation in Plants Containing SSCpro:TAG1

Next, we tried to increase oil content using the *SSC* promoter. Because *TAG1* encoding DGAT1 is one of the good candidates to express in seeds to increase oil content [11,12], we used the *AtTAG1* gene first. The *AtTAG1* gene was ligated to the *SSC* promoter and introduced into *Arabidopsis*. For comparison, we also made transgenic plants carrying *FAE1pro*:*TAG1*. The oil content of mature seeds from 11 independent transgenic plants was measured.

**Figure 3:**
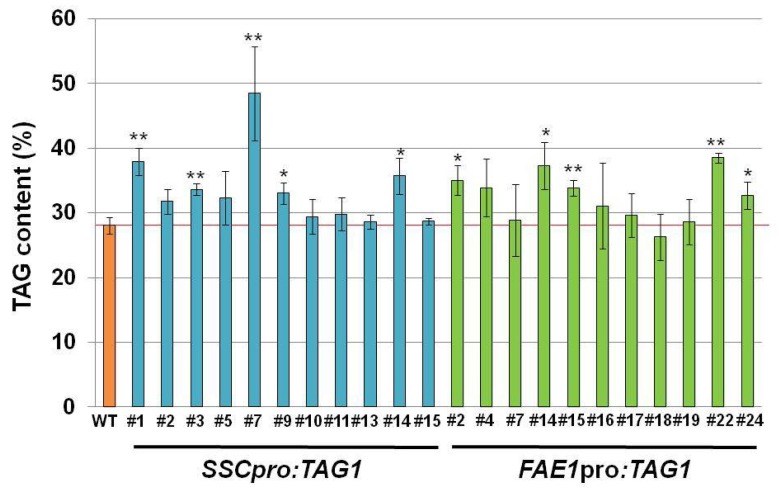
Triacylglycerol (TAG) content in seeds. TAG content in mature seeds from independent transgenic lines expressing *AtTAG1* under control of the *seed-specific chimeric* (*SSC*) promoter or *fatty acid elongase 1* (*FAE1*) promoter. TAG content is expressed as a percentage of mature seed weight. Values are means ± standard deviation of measurements of 3–5 independent experiments. Statistical analysis carried out by Student’s *t*-test. Asterisks indicate a significant difference between the wild-type (WT) and transgenic lines at *p* < 0.01 (**) and *p* < 0.05 (*).

The oil content in both transgenic plants varied depending on the lines (Figure 3). In both transgenic plants, at least five plants contained significantly increased levels of oil compared with the WT (*SSCpro:TAG1* plants #1, #3, #7, #9 and #14, and *FAE1pro:TAG1* plants #2, #14, #15, #22 and #24). Although oil accumulated to similar levels in both *SSCpro:TAG1* and *FAE1pro:TAG1*, *SSCpro:TAG1* #7 accumulated much higher levels of oil (72% increase compared with WT). The highest oil content among *FAE1pro*:*TAG1* plants (#22) was increased by 37% compared with WT.

**Figure 4:**
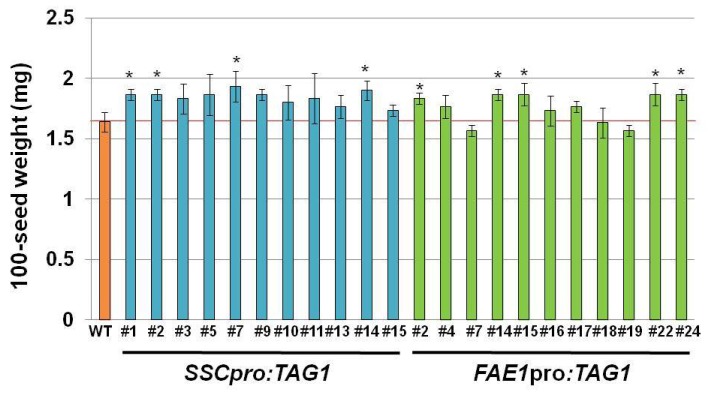
Seed weight of independent transgenic lines. Seed weight of independent transgenic lines expressing *TAG1* under control of the *SSC* promoter or *FAE1* promoter. Values are means ± standard deviation of three independent measurements of the weight of 100 seeds. Statistical analysis carried out by Student’s *t*-test. Asterisks indicate a significant difference between the WT and transgenic lines at *p* < 0.05 (*).

The average weights of 100 seeds from all of *SSCpro:TAG1* were heavier than that of WT (Figure 4). Therefore, oil contents per seed in plants carrying *SSCpro:TAG1* were higher than WT, while some *FAE1pro:TAG1* plants (#7, #18 and #19) showed lower oil contents than WT (Figure S1). The TAG content of *SSCpro:TAG1* #7 per seed was doubled, and that of *FAE1pro:TAG1* #22 (the highest oil content among *FAE1pro:TAG1* plants) was increased by 56% compared with WT.

**Figure 5:**
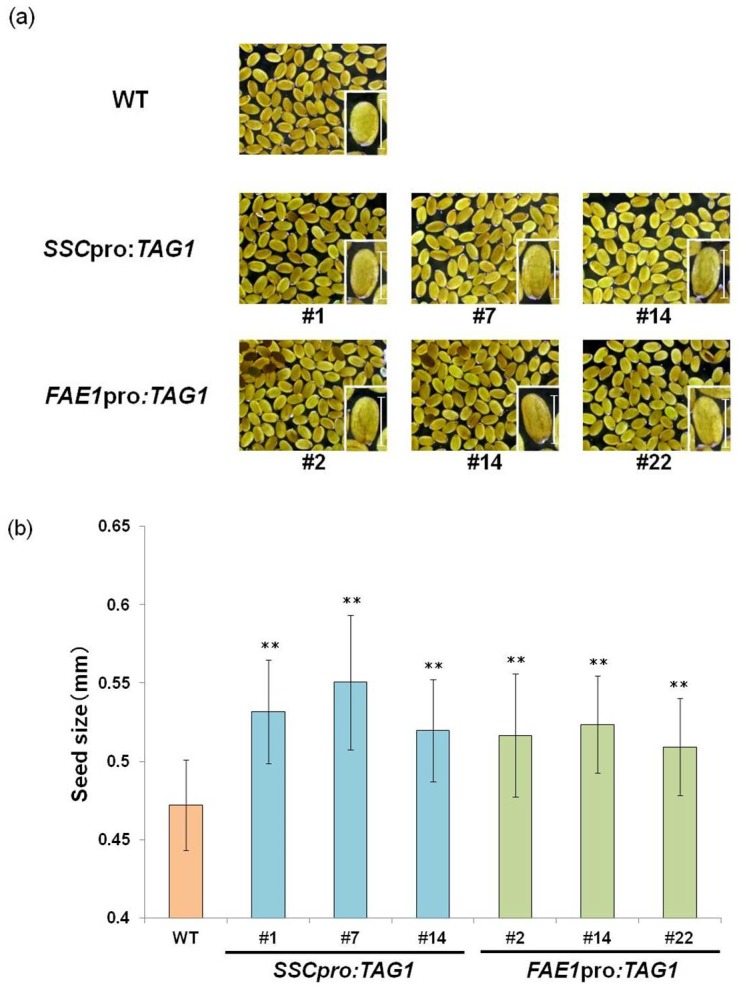
Seed size of transgenic plants. (**a**) Images and (**b**) the length of seeds that accumulated the three highest amounts of TAG among transgenic lines expressing *TAG1* under control of the *SSC* promoter or *FAE1* promoter. Scale bars = 0.5 mm. Values are means ± standard deviation of measurements of 50 seeds. Asterisks indicate a significant difference between the wild-type (WT) and transgenic lines at *p* < 0.01 (**).

The seed size of transgenic seeds that contained the three highest amounts of oil (*SSCpro:TAG1* plants #1, #7 and #14, and *FAE1pro:TAG1* plants #2, #14 and #22) was measured. All of these seeds were significantly larger than WT seeds (Figure 5).

**Figure 6:**
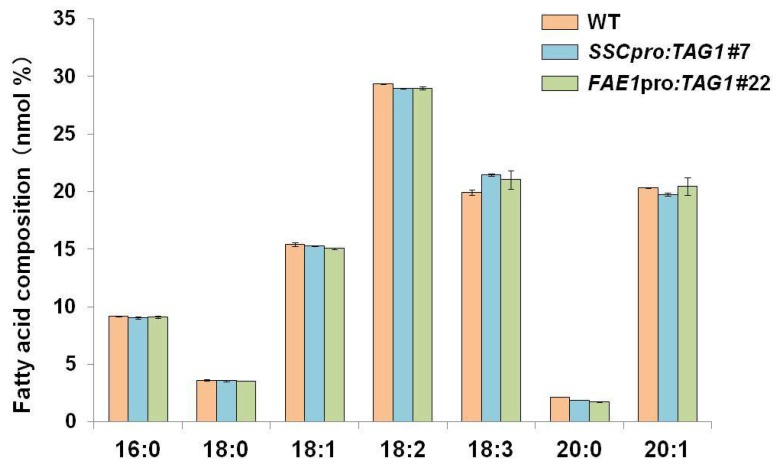
Fatty acid (FA) composition in seeds from transgenic plants. As representatives, FA compositions of seeds from transgenic lines that accumulated the highest amounts of TAG; lines *SSCpro:TAG1* #7 and *FAE1:TAG1* #22 are shown compared with that of WT seeds. Values are means ± standard deviation of measurements of 3 independent experiments.

The fatty acid composition in seeds form *SSCpro:TAG1* and *FAE1pro*:*TAG1* plants was analyzed. There was no notable difference in fatty acid composition in both *SSCpro:TAG1* and *FAE1pro*:*TAG1* plants compared with the WT. Figure 6 shows a representative example of the fatty acid composition of *SSCpro:TAG1* #7 and *FAE1pro:TAG1* #22, which accumulated the highest amounts of oil in each transgenic line.

### 2.4. TAG1 Gene Expression in Transgenic Plants

**Figure 7:**
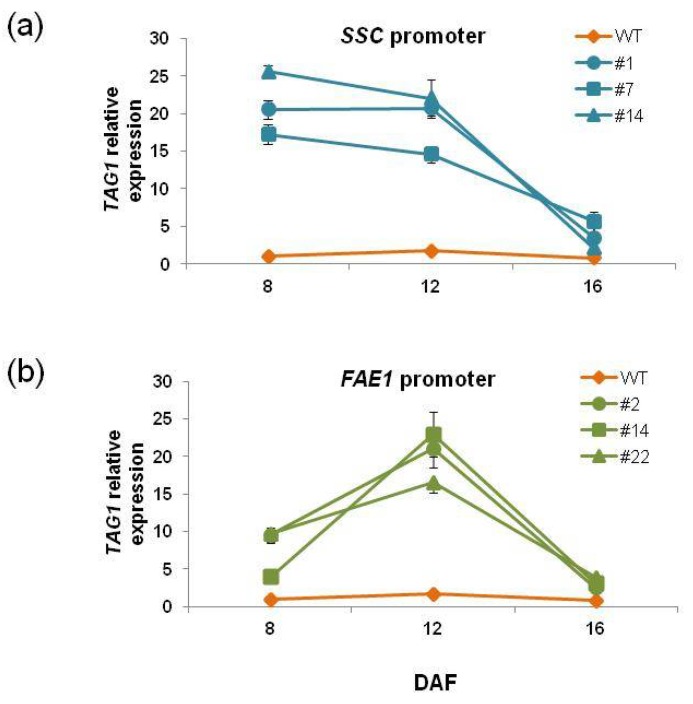
*TAG1* expression in seeds from transgenic plants. The expression levels of *TAG1* in seeds at 8, 12 and 16 days after flowering (DAF) in (**a**) *SSCpro:TAG1* and (**b**) *FAE1pro:TAG1* seeds were quantified by quantitative reverse transcription–polymerase chain reaction (qRT–PCR) and presented as relative values versus the level in WT seeds at 8 DAF. The gene expression levels were normalized using *ELONGATION FACTOR1 αA4* (*EF1αA4*). Data shown represent the mean ± standard deviation of 3 (seeds at 8 DAF) and 5 (seeds at 12 and 16 DAF) biological replicates.

We examined the expression level of *TAG1* in transgenic plants that accumulated higher levels of oil. *SSCpro:TAG1* plants #1, #7 and #14, and *FAE1pro:TAG1* plants # 2, #14 and #22, were analyzed. Seeds at 8, 12 and 16 DAF were collected, and expression levels were measured by quantitative reverse transcription–polymerase chain reaction (qRT–PCR). Figure 7 shows the relative expression level of *TAG1* in each sample relative to the level of seeds from WT plants at 8 DAF, which was taken as 1. In WT seeds, *TAG1* expression was increased in seeds at 12 DAF (1.7-fold higher than seeds at 8 DAF) and then decreased in seeds at 16 DAF to similar levels as in seeds at 8 DAF.

In *SSCpro:TAG1* plants, the highest expression was observed in seeds at 8 DAF, from 16 to 26-fold higher than WT seeds, but expression was slightly decreased in seeds at 12 DAF. Although the expression was decreased in seeds at 16 DAF, the expression was still higher than that of WT seeds (relative value from 2.1 to 5.6-fold). On the other hand, *FAE1pro:TAG1* plants showed the highest *TAG1* expression in seeds at 12 DAF (relative value from 16 to 23-fold). The expression in both seeds at 8 DAF and 16 DAF was also higher than WT seeds (relative value from 4 to 10-fold in seeds at 8 DAF and from 2 to 4-fold in seeds at 16 DAF).

These results indicated that much higher *TAG1* expression levels in seeds at 8 DAF in *SSCpro:TAG1* plants than *FAE1pro:TAG1* plants were not related to the amount of oil accumulation because the oil levels in these transgenic plants except for *SSCpro:TAG1* #7 were similar. *TAG1* expression levels in seeds at 8 and 12 DAF in *SSCpro:TAG1* plant #7, which accumulated the highest level of oil in seeds, was lowest among the three transgenic plants, whereas the expression in seeds at 16 DAF was highest.

## 3. Discussion

We constructed a chimeric promoter that showed strong activity from early to late stages of seed development by fusing the 5′-UTR of *AtBCCP2* to a 1.2 kb region from the TIS of *AtFAE1* gene (Figure 1 and Figure 2).

Transgenic plants carrying *SSCpro:TAG1* showed increased amounts of storage oil in seeds compared with WT plants. Compared with plants carrying *FAE1pro:TAG1*, similarly increased levels of seed oil were seen in *SSCpro:TAG1* plants (Figure 3). However, one line containing *SSCpro:TAG1*, #7, accumulated much more oil in seeds than the other transgenic plants (Figure 3). The seed weight and seed size in plant line #7 was also heavier and larger than other seeds. Because we used transgenic plants carrying a single copy transgene, which was confirmed by segregation analysis and quantitative PCR, the effect of copy number on the expression of transgene can be excluded.

Expression of the *TAG1* gene in transgenic plants showed a unique pattern (Figure 7). *TAG1* expression in *SSCpro:TAG1* plants at 8 DAF was much higher than that of WT plants. The level was slightly decreased at 12 DAF, and then at 16 DAF, the *TAG1* expression level was lowest among the timepoints measured. On the other hand, *TAG1* expression was highest at 12 DAF in *FAE1pro:TAG1* plants.

We could not find a correlation between oil content and *TAG1* expression level at 8 DAF and 12 DAF. However, it appeared that there was a correlation between oil content and the expression level at 16 DAF. The *SSCpro:TAG1* plant line with highest oil contents (#7) showed the highest expression level of *TAG1* at 16 DAF (relative value 5.6 times), and plant line #14, which accumulated the lowest oil content among the three transgenic lines carrying *SSCpro:TAG1*, showed the lowest expression of *TAG1* at 16 DAF (relative value 2.1-fold). Similarly, in plants carrying *FAEpro:TAG1*, *TAG1* expression at 16 DAF showed a correlation with oil content; the *TAG1* expression relative value of plant line #22, which had the highest oil content, was 4.0-fold and that in plant line #2, with the lowest oil content, was 2.4-fold. The results indicated that higher expression levels of the *TAG1* gene at 16 DAF (late stage of seed development) were important for higher oil content. In this respect, high expression of the *TAG1* gene in the early stage in *SSCpro:TAG1* plants was not advantageous to accumulate higher content of TAG. However, the chimeric promoter showed high expression in the late stage of seed development, which the *FAE1* promoter could not produce (Figure 2 and Figure 7). Therefore, transgenic plants with ideal expression levels of *TAG1*, such as plant line #7 (Figure 7), which accumulate much more oil than other transgenic plants, could be produced by using the chimeric promoter.

The timing of gene expression is important. Kanai et al. (2015) [15] showed that the *AtWRI1* gene under control of the *FUSCA3* (*FUS3*) promoter, which is activated during the middle phase of seed development (peaking at around 8 DAF), enhanced *Arabidopsis* seed oil content much more than that under the control of the *35S* promoter or the *LEA* promoter, which is activated in the late phase of seed development (increases from >8 DAF). Previous studies also showed that activation of *WRI1* during the late phase of seed development was not effective in increasing oil content [5,20]. Because fatty acids are essential not only for oil synthesis but also for membrane biogenesis, which is required for embryo development, fatty acid synthesis probably has to be largely activated during the early-to-middle phase of seed development. Moreover, if there is competition between oil synthesis and protein synthesis for carbon resources during seed development, enhancement of fatty acid synthesis should be started before protein synthesis starts to increase. On the other hand, activation of the *TAG1* gene in the late phase of seed development successfully increased oil content [11,12]. *TAG1* encodes DGAT1, which catalyzes the last step of TAG synthesis and is originally expressed in the later stage of seed development in *Arabidopsis* [21]. Therefore, later expression is probably more effective for the *TAG1* gene to enhance oil production.

In this study, we succeeded in increasing seed oil content using a constructed chimeric promoter. However, expressing genes for enzymes or transcription factors involved in fatty acid synthesis, such as genes for WRI1 or homomeric ACCase (cytosolic ACCase or ACCase from Garmineae), which play a pivotal role in fatty acid synthesis, can take advantage of the properties of the *SCC* promoter fully because high expression of these genes in the early stage of seed development is required for high accumulation of TAG.

Because the promoter contains the WRI1 binding site and the *FAE1* promoter, which is reported to be regulated by B3 domain factors, LEAFY COTYKEDON 2 and FUS3, through the RY motif [22], we can use the chimeric promoter in other plants that have similar transcriptional regulation of seed development to *Arabidopsis*. Brassicaceae, including *Camelina sativa* and *Brassica napus*, may be good target plants to be manipulated using the chimeric promoter. Expression of target genes in novel expression patterns could be a promising strategy for substance production in seeds or research on seed development.

## 4. Materials and Methods

### 4.1. Plant Materials and Growth Conditions

The surfaces of seeds were sterilized in 20% bleach for 15 min, rinsed five times with sterile water, and then plated on half-strength Murashige and Skoog (1/2 MS) medium, pH 5.7, 0.8% agar, and 1% sucrose. The transgenic plants were screened on the 1/2 MS with kanamycin (30 μg mL^−1^). The seeds from homozygous transgenic and WT plants were placed on 1/2 MS without kanamycin. Plates were first placed at 4 °C for 2 days and then transferred to a growth chamber at 23 °C with a light period of 16 h (65 μmol m^−^^2^ s^−1^) and a dark period of 8 h. Seedlings were transferred to soil at 7–10 days after germination.

To collect developmental-stage siliques, colored threads were used to tag flowers on the day of flowering (day 0) when petals just appeared. The siliques that developed from the tagged flowers were collected at specific time points.

For measurement of oil content in seeds, mature seeds were collected from homozygous transgenic T3 plants which were planted at same time and grown under the same condition.

### 4.2. Construction of Binary Vector and Transformation

Primers used in PCR reactions are described in Table 1. The 5′-UTR fragment of the *AtBCCP2* gene was amplified using primers BCCP2XF/BCCP2SlR. The promoter of the *AtFAE1* gene was amplified using primers FAEp2kSalF/FAEpSalR. The PCR products were cloned into pCR-Blunt (Invitrogen, Carlsbad, CA, USA). The 5′-UTR fragment of the *AtBCCP2* was digested with *Xho*I and *Sal*I, and the *FAE1* promoter was digested with *Sal*I and *Hin*dIII whose site is located 1.2 kb upstream of the TIS of the *AtFAE1* gene. Each fragment was collected and ligated to the *Hin*dIII/*Sal*I site of pBI101 to construct *SSCpro:GUS*. To construct *FAE1pro:TAG1*, *Hin*dIII/*Sal*I fragment of the *FAE1* promoter was ligated to the *Hin*dIII/*Sal*I site of pBI101.

In order to increase the restriction sites to insert the *TAG1* gene, the *GUS* gene was replaced by the *GFP* gene with several restriction sites on both the 3′ and 5′ sites. The *GFP* gene was amplified using primer set GFPXXHF/GFPHSSR and the amplified fragment was digested with *Sal*I and *Sac*I. The *SSCpro:GUS* vector was digested with *Sal*I and *Sac*I and the *GUS* gene was replaced by the *GFP* fragment (*SSCpro:GFP*). The *TAG1* fragment was amplified using primer set TAG1XbaSmaF/TAG1EcoRVR and digested with *Xba*I/*Eco*RV, and was ligated into the *Xba*I/*Sma*I sites of the *SSCpro:GFP* vector to construct *SSCpro:TAG1*.

*Arabidopsis thaliana* (ecotype Col-0) was transformed with *Agrobacterium tumefaciens* (strains GV3101) using the floral dip method [23]. Several transgenic lines were screened on 1/2 MS with kanamycin (30 μg mL^−1^) and homozygous transformant lines were established from isolated independent transgenic lines. We confirmed the introduced gene by PCR using a primer set TAG1XbaSmaF/TAG1EcoRVR.

### 4.3. GUS Staining Assay and GUS Fluorometric Assay

Histochemical staining of GUS activity was conducted using transgenic plants as described previously [16]. Fresh tissue was incubated in reaction solution (1 mM X-Gluc, 50 mM sodium phosphate buffer pH 7.0, 1 mM potassium ferricyanide/ferrocyanide mixture, 0.01% Triton X-100, 10 mM 2-mercaptoethanol, 20% methanol, 1 mM EDTA) at 37 °C for 12 h. After staining, the tissue was fixed in a mixture of ethanol:acetic acid at a 6:1 ratio and rinsed with 70% ethanol. Tissues were observed and photographed using a dissecting microscope.

GUS activity in protein extracts was measured using the fluorogenic substrate 4-methylumbelliferyl b-d-glucuronide (MUG) as described previously [16]. Proteins were extracted from seeds at 8, 12, and 16 DAF (more than five independent plants). Total protein extracts were prepared by grinding the tissues in extraction buffer (50 mM sodium phosphate, pH 7.0, 10 mM EDTA, 10 mM β-mercaptoethanol) containing 0.1% (*w*/*v*) SDS and 1% Triton X-100, followed by centrifugation at 13,000× *g* for 10 min. GUS activity in the supernatants was measured in extraction buffer containing 1 mM MUG and 20% methanol. Reactions were stopped by adding 0.2 M Na_2_CO_3_, and the amount of 4-methylumbelliferone was calculated by relating relative fluorescence units with those of a standard of known concentration. The protein concentration of extracts was determined using Protein Assay Kits (Bio-Rad, Hercules, CA, USA).

### 4.4. Measurement of Oil Content

Lipids were extracted in accordance with the Folch method [24] with some modifications.

A total of 10 mg of seeds was ground in a glass tube, and 2 mL of chloroform:methanol (1:1, *v*/*v*) was added. After standing at room temperature for 30 min, 1 mL of 0.9% NaCl solution (*w*/*v*) was added and mixed by vortexing. The extract was centrifuged at 845× *g* for 15 min at 4 °C. The lower layer was transferred to a new tube. Again, 1 mL of 0.9% NaCl solution (*w*/*v*) was added to the collected extract and mixed by vortexing. The extract was centrifuged at 845× *g* for 5 min at 4 °C. The same process was repeated once again. Finally, the lower layer was transferred to a glass vial and the extract was dried and dissolved in 100 μL of chloroform. A 20 μL aliquot of lipid sample was spotted onto a silica gel thin-layer chromatography (TLC) plate (Merck Millipore, Darmstadt, Germany) to separate TAG from total lipid. TLC separation was carried out using petroleum ether:diethyl ether:acetic acid (70:30:1). The TAG spot was transferred to a glass tube, and 1 mL of hydrogen chloride methanol solution was added and heated at 80 °C for 30 min. After cooling, 1 mL hexane and 1 mL 0.9% NaCl solution was added and mixed by vortexing. The sample was centrifuged at 845× *g* for 5 min at 4 °C and 800 μL of supernatant was transferred to a new tube. The collected solution was dried and the collected fatty acid methyl ester was dissolved in 50 μL of hexane. The fatty acid methyl esters were quantified using gas–liquid chromatography (GC2014, Shimadzu, Kyoto, Japan) using a capillary column (BPX-90, SGE Analytical Science, Ringwood, VIC, Australia).

### 4.5. Expression Analysis

Total RNA was isolated from seeds at 8, 12, and 16 DAF using TriPure (Roche Diagnostics, Mannheim, Germany) and Fruitmate (TAKARA, Ohtsu, Japan). cDNA was synthesized using 0.5 μg of RNA as a template and ReverTra Ace (TOYOBO, Ohsaka, Japan).

Real-time PCR amplification of cDNAs was conducted using a LightCycler 480 (Roche Diagnostics) in a 384-well PCR plate. The reaction was carried out in a 10 μL reaction volume containing 5 μL SYBR Premix Ex Taq II (TAKARA) with 0.2 μM each of the forward and reverse primers and 1 μL cDNA (10-fold dilution). The primer sets used for real-time PCR are shown in Table 1. The primers for amplification of *TAG1* were synthesized according to Yang et al. (2011) [25]. Expression levels of *ELONGATION FACTOR1 αA4* (*EF1αA4*) were used for signal normalization of real-time PCR. The *EF1αA4* primers were designed by the Universal ProbeLibrary Assay Design Center (Roche Diagnostics). All reactions were performed independently at least five times, and at least three sets of consistent data were used for analyses. Relative gene expression levels were calculated using the 2^−ΔΔCT^ method [26]. To validate the reliability of the data, amplification efficiencies between the target genes and the house-keeping genes of all the real-time PCR reactions were compared, and dissociation curves of all PCR products were examined to ensure the quality of the PCR.

## Figures and Tables

**Table 1:** Primers used in this study.

	Name	Sequence
**Construction of Chimeric Promoter**		
*BCCP2 5'-UTR*	BCCP2X F	GCGCTCGAGACAAAAGGAGCGGTTTTGG
	BCCP2Sl R	GCGGTCGACTGACGCCATTGTTGAGAC
*FAE1*	FAEp2kSal F	CGTCGACGGATCCCGGATTCTATTCACTCTATC
	FAEpSal R	GGTCGACTCTGTTTGTGTCGGAAAATAATGG
*GFP*	GFPXXH F	CTCGAGTCTAGAAAGCTTATGGGTAAGGGAGAAGAAC
	GFPHSS R	GAGCTCCCCGGGAAGCTTTTATTTGTATAGTTCATCC
*TAG1*	TAG1XbaSma F	TCTAGACCCGGGATGGCGATTTTGGATTCTG
	TAG1EcoRV R	GCGATATCTCATGACATCGATCCTTTTCG
**qRT-PCR**		
*TAG1*	AtDGAT1 F	AATGTGGAATATGCCTGTTCATAAA
	AtDGAT1 R	CCCACCGTTGAGCCAAACC
*EF1αA4*	Real-time EF1aA4 F	CTTGGTGTCAAGCAGATGATTT
	Real-time EF1aA4 R	CGTACCTAGCCTTGGAGTATTTG

## Supplementary Materials

Supplementary materials can be found at http://www.mdpi.com/1422-0067/19/6/1667/s1.
