# Supplementary material for: Design of a Seed-Specific Chimeric Promoter with a Modified Expression Profile to Improve Seed Oil Content
Source: Int J Mol Sci. 2018 Jun 5;19(6):1667. doi: 10.3390/ijms19061667 (PMC6032214; doi:10.3390/ijms19061667)
Supplement: Supplementary file 1 [file ijms-19-01667-s001.pdf]

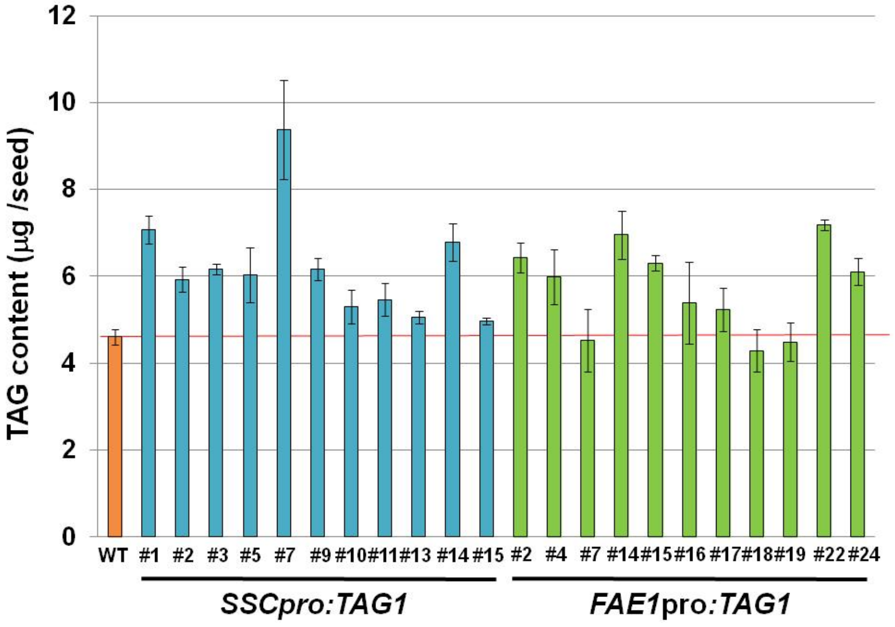

Figure S1. Triacylglycerol (TAG) content per seed. TAG content in seeds from independent transgenic lines expressing *TAG1* under control of the *SSC* promoter or *FAE1* promoter. TAG content is expressed as weight (μg) per seed. Values are means ± standard deviation of measurements of 5 independent experiments.
